# Supplementary material for: Emerging patterns of plasmid-host coevolution that stabilize antibiotic resistance
Source: Sci Rep. 2017 Jul 7;7:4853. doi: 10.1038/s41598-017-04662-0 (PMC5501780; doi:10.1038/s41598-017-04662-0)
Supplement: Supplementary file 1 — Supplementary Methods, Tables, Figures [file 41598_2017_4662_MOESM1_ESM.pdf]

SUPPLEMENTARY INFORMATION

Emerging patterns of plasmid-host coevolution that stabilize antibiotic resistance.

Thibault Stalder, Linda M. Rogers, Chris Renfrow, Hirokazu Yano Zachary Smith and Eva M. Top

Supplemental Methods: p. 1-2

Supplemental Tables: p. 3-4

Supplemental Figures: p. 5

Supplemental Methods

Use of the BIC to compare plasmid persistence profiles

The plasmid population dynamics model described by De Gelder *et al*[1] and Ponciano *et al.*[2] is a mechanistic model that estimates the 3 main parameters affecting the persistence of a conjugative plasmid, namely the frequency of plasmid loss (λ), the fitness cost of plasmid carriage (σ), and the conjugation transfer frequency (γ). The unit of time in this model is one generation of plasmid carrying cells. Hence, from one generation to the next, the number of plasmid carrying cells at generation t, n_t simply doubles. However, during that same time period a plasmid carrying cell can lose its plasmids via segregation with probability λ. Hence, after one generation the expected number of plasmid carrying cells will be $2(1-\lambda)n_t$. During the same time period (one generation), the plasmid-free cells multiply at a rate of $2^{1+\sigma}$, where σ is the fitness advantage of not carrying the plasmid (i.e. plasmid cost). Besides growth, the numbers of both types of cells can change due to conjugation. In the model, plasmid carrying cells are gained by a conjugation frequency γ that is modulated by the fraction of available plasmid receivers and donors. After writing the model and analyzing its predictions, Ponciano *et al.*[2] reduced the two-

dimensional model (i.e. a model with one equation for the plasmid-free cells and one equation for the plasmid carrying cells), to a one-dimensional model that follows the fraction of plasmid-free cells. The predictions of the changes in the proportion of plasmid-free cells then can be used as the model-generated probabilities of sampling a given fraction of plasmid-free cells. These predictions are then used to estimate the model parameters via a binomial regression-like model and maximum likelihood. To fit these models we used the beta test version of an R-package that is available at https://github.com/jmponciano/StabilityToolkit/blob/master/RunningStabToolsPack.zip. The package implements the calculations in De Gelder *et al*[1] and Ponciano *et al.*[2]. These references should be consulted for a full account of the statistical details of the model fitting process.

To determine if the plasmid persistence profile of two sets of clones were similar or not, the values for λ, σ and γ were assigned to each set of plasmid persistence profiles to be compared. To determine if the persistence dynamics of two bacteria-plasmid pairs where similar or not we used a widely-used statistic to perform model selection, the Bayesian Information Criterion (BIC) [3,4].

1. De Gelder, L. *et al.* Combining mathematical models and statistical methods to understand and predict the dynamics of antibiotic-sensitive mutants in a population of resistant bacteria during experimental evolution. *Genetics* **168,** 1131–1144 (2004).

2. Ponciano, J. M., De Gelder, L., Top, E. M. & Joyce, P. The population biology of bacterial plasmids: a hidden Markov model approach. *Genetics* **176,** 957–968 (2006).

3. Burnham, K. P., Anderson, D. R. & Burnham, K. P. *Model selection and multimodel inference: a practical information-theoretic approach*. (Springer, 2002).

4. Loftie-Eaton, W. *et al.* Evolutionary paths that expand plasmid host-range: implications for spread of antibiotic resistance. *Mol. Biol. Evol.* **33,** 885–897 (2016).

Table S1: Summary of the mutations in the plasmid pBP136Km and the *S. oneidensis* MR-1 host genome (NC_004347) and native plasmids.

Location	Gene	Position	L1-1	L1-2	L1-3	L4-1	L4-2	L4-3	Type	Description
pSMR-1	*SOA0171-SOA0170*	1		x					Deletion	Plasmid loss
pBP136	*trfA*	1002					x	x	Dulpication	DNA-binding protein for plasmid replication
pBP136	*trfA*	1010	x	x	x				Duplication	DNA-binding protein for plasmid replication
pBP136	*oriV / trfA*	42053-42059						x	Insertion	Insertion of transposon Tn*6374*
NC004347	*SO 0208*	215716						x	Q130H (CAA>CAC)	RNA-binding protein CDS
NC004347	*SO 0798* / SO 0799*	812695	x	x	x'				Intergenic (-46/-104)	TonB dependent receptor/protein of unknown function DUF985
NC004347	*SO 0798* / SO 0799*	812704				x	x'	x	Intergenic (-55/-95)	TonB dependent receptor/protein of unknown function DUF985
NC004347	***fur***	**2042240**				x			**A53T (GCA>ACA)**	**transcriptional repressor of iron homeostasis Fur**
NC004347	*hmgR / SO 1966*	2071070				x	x	x	Intergenic (+103/-46)	homogentisate responsive transcriptional repressor of homogenetisate degradation HmgR/protein of unknown function DUF124
NC004347	*SO 2050*	2151022						x	Q17H (CAA>CAC)	protein of unknown function DUF938
NC004347	*fnr*	2464194						x	L28R (CTT>CGT)	oxygen responsive transcriptional regulator of anaerobiosis response Fnr
NC004347	*ackA / pta*	3047125						x	Intergenic (+73/-49)	acetate kinase AckA/phosphate acetyltransferase Pta
NC004347	***tdk / dcp***	**3273867**				x			**intergenic (-261/-275)**	**thymidine kinase Tdk/Putative gene (NON-annotated)**
NC004347	*SO 3268*	3405587	x	x	x				P167R (CCA>CGA)	flagellin modification glycoside hydrolase family 57
NC004347	*tnpA / tnpA*	3732282						x	intergenic (-292/-67)	ISSod3 transposase TnpA ISSod3/ISSod1 transposase TnpA ISSod1
NC004347	*SO 3627 / SO 3629*	3787790	x						intergenic (+334/476)	transcriptional repressor of flavocytochrome c TetR family/N terminal BluF domain containing protein
NC004347	*SO 3793 / SO 3794*	3945110						x	intergenic (-63/-85)	cupin 2 conserved barrel domain containing protein/morn variant repeat protein
NC004347	*murA*	4094448						x	L88V (TTA>GTA)	UDP N acetylglucosamine 1 carboxyvinyltransferase MurA

*, Genes whose expression has been shown to be under regulation of the Fur protein. In bold are the unique mutations found in L4-1; ', mutations did not pass the breseq 0.25d filters but where found to be present when manually verifying the alignments.

Table S2: Primers used for the endogenous plasmid/transposon junction amplification.

Primer ID	Sequence (5’-3’)
1568_R (A)	CGCCAAGATAGGTTCAGGTAAA
1561_F (B)	GTGTCGGTTCCTACGCTATA
1562_F (C)	ACGAGAAATGTCAGGGTTAAGG
1569_R (D)	TCCCGGTTAACTCTTTCTTACC
1564_F (E)	ATGCGACTGTATGAGCCAAG
1570_R (F)	GGGCTTGAGTATCCGTTCTATT

Table S3: Expected and estimated length of the amplicons resulting from PCR reactions that targeted the transposon junction in *S. oneidensis* MR-1. Primer pairs are described in Table S2.

Primer pair	Expected PCR product		PCR product estimated size
	1 plasmid scenario	2 plasmids scenario	
A:C	652	652	650*
B:F	696	696	700*
A:E	158 / 790	158 / 790	200* / 800*
B:D	658	658	650*
C:E	-	-	-
D:E	5056 / 5688	-	-
C:F	5588	-	-
D:F	-	-	-
C:D	24,806	5550	5500*
E:F	-	7000 / 7726	**650*** / 5000* / 5500*

-: No PCR product expected or detected; *: specificity of the PCR product to the endogenous plasmid confirmed by Sanger sequencing. Note: the unexpected 650-bp fragment corresponded to a version of the small plasmid without the transposon

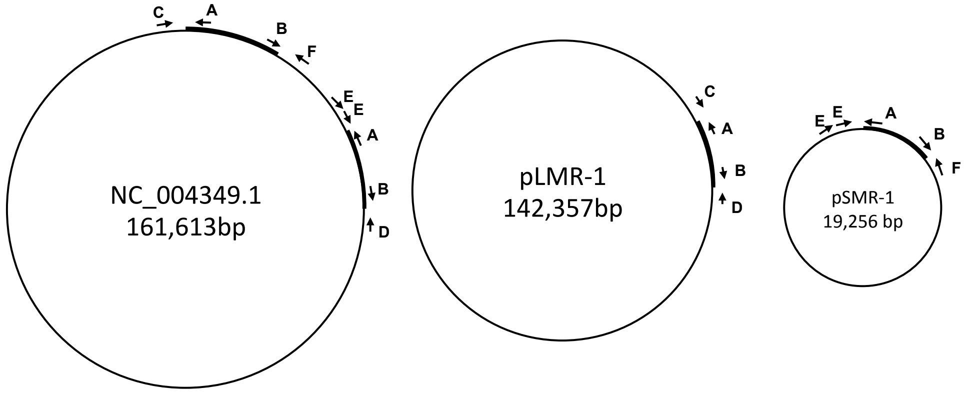

Figure S1: Plasmid maps describing the targets of the primers used to amplify the transposon junctions. The transposon Tn*6374* is represented in bold. The NC_004349.1 map is based on the published Genbank sequence, and represents the scenario where the endogenous plasmid is one single replicon and the pLMR-1 and pSMR-1 maps represents the scenario where the endogenous plasmids are two independent replicons.

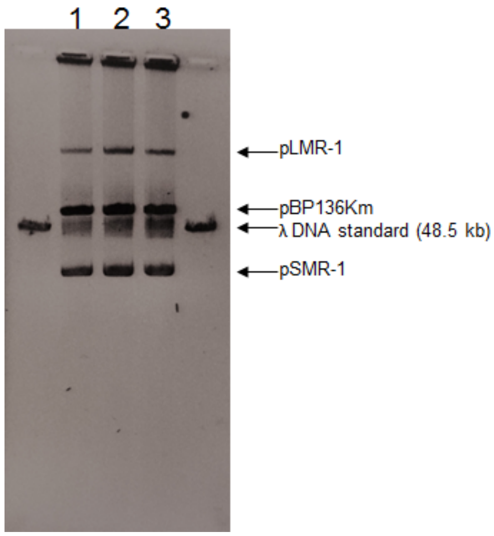

Figure S2: Example of electrophoresis using agarose gel (0.8%) of triplicate plasmid DNA samples extracted from MR-1 (pBP136Km) (Lanes 1, 2 and 3). The far left and right lanes contain undigested lambda DNA.
